# Teaching medicine with the help of “Dr. House”

**DOI:** 10.1371/journal.pone.0193972

**Published:** 2018-03-13

**Authors:** Andreas Jerrentrup, Tobias Mueller, Ulrich Glowalla, Meike Herder, Nadine Henrichs, Andreas Neubauer, Juergen R. Schaefer

**Affiliations:** 1 Center for Unknown and Rare Diseases, UKGM GmbH, University Clinic Marburg, Philipps-University, Marburg, Germany; 2 Instruction and Interactive Media Research Group, Department of Psychology and Sport Science, Justus Liebig University, Giessen, Germany; 3 Department of Psychosomatic Medicine and Psychotherapy, Klinikum Leer gGmbH, Leer, Germany; 4 Department of Therapeutic Pedagogy and Special Education, Justus Liebig University, Giessen, Germany; 5 Internal Medicine–Hematology, Oncology and Immunology, UKGM GmbH, University Clinic Marburg, Philipps-University, Marburg, Germany; Monash University, AUSTRALIA

## Abstract

TV series such as “House MD”, “Grey´s Anatomy” or “Emergency Room” are well perceived by medical students. Seminars featuring medical TV series such as “House MD” might serve as door-opener to attract medical students to learn more about rare diseases. The TV series “House MD” is troublesome for the main character Dr. House is an excellent diagnostician but at the same time a rather misanthropic person. Therefore, lecturing medicine with the help of “House MD” requires constant evaluation. From 2008 to 2016 we are using the well-known TV series “House MD” continuously to attract medical students and teach them about rare diseases as well as diagnostic strategies. We collected from 213 students a detailed questionnaire assessing their learning experience. 76.6% of our students (n = 157) reported to watching medical dramas on a regular basis. The Dr. House seminar was compared to traditional seminars and our students reported an improved learning effect (69.9%), better concentration (89.7%), higher motivation to participate (88.7%), and more fun (86.7%) (all p<0.001). The students see Dr. House’s behavior quite critically. Likert assessment on a 5-point scale identified strong disagreement with Dr. House´s interpersonal skills in dealing with his colleagues (median = 1) and patients (median = 1). At the same time, the students strongly agreed with his outstanding diagnostic (median = 5) and therapeutic capabilities (median = 4). Medical students visiting a Dr. House teaching seminar are highly motivated to learn more about rare diseases. They were positively influenced by TV series such as Dr. House to improve their diagnostic and clinical skills. At the same time, they are critical enough not to see Dr. House as a role model for their own personality. Well performed medical TV shows such as Dr. House can successfully be used in an educational setting to motivate medical students to come into seminars to learn more about rare diseases.

## Introduction

In recent years, medical dramas such as “Emergency Room”, “Grey´s Anatomy” or “House MD” found a broad viewership in the TV-watching western world. These series not only deliver pure entertainment but also provide some knowledge on diseases. “House MD” is of special interest since the authors of this show try to describe unusual cases and rare diseases. In addition, “House MD” is one of the best-known US-American television medical dramas. “House MD” has been co-created by David Shore and Paul Attanasio. The TV show debuted in the USA on the Fox network on November 16, 2004 and ended on May 21, 2012. According to the Guinness Book of Records “House MD” became one of the most successful TV drama on the globe reaching 81.8 million people in 66 different countries [1]. Also, the program was critically acclaimed and received several awards, including a Peabody Award, two Golden Globe Awards, and three Primetime Emmy Awards. “House MD” reached high viewership ratings around the world, also by the medical community, especially by medical students. A recent survey of nearly 400 medical and nursing students revealed that 76% of doctors in training watched "House MD" and 85% of medical students said they had watched a medical drama in the previous year [2].

A special feature of “House MD” is the fact that the themes usually report on rather rare diseases, many of them had been published in former case report sections of journals such as NEJM, THE LANCET, BMJ or others. Most importantly to us, the reported symptoms are fairly well described since the writers are supported by an excellent team of medical experts. They try hard to include scientifically correct medical information along the themes–of course within the limitations of a TV show that is supposed to entertain the audience in the first place [3]. Since “House MD” depicts both the practice of medicine and bioethical issues in a strikingly realistic but sometimes inaccurate fashion, we decided to use this TV series for educational purposes. Our intention was on one side to engage our students in critical thinking on the medical and bioethical content in “House MD” episodes. On the other side, we used the success of “House MD” as a door-opener to attract our students’ attention for rather rare and unusual diseases. Therefore, we started in autumn 2008 a clinical lecture class called “Dr. House revisited–or: would we have saved the patient in Marburg* as well?” (*Marburg is the town where our university clinic is located) based on the “House MD” TV series. As expected these seminars were of high interest to our students. Unexpectedly this seminar was also of great interest for public media such as newspapers, radio and TV news. Obviously, the public media was convinced that we would produce in Marburg, the oldest protestant university worldwide, “little Dr. Houses”,—a concern which was needless as we will demonstrate,—luckily.

## Materials and methods

### Legal aspects using copyright-protected media

Before starting this project and using copyright-protected media for teaching purposes we had to clarify some potential legal issues. In our case, we had to follow the German law for intellectual rights = Urheberschutz; UrhG § 52 a), which would permit us to use only small parts of this TV show for non-profit lecturing in small, closed teaching groups. In addition, we informed the national right owners (RTL Köln, Germany; Universal Picture, Hamburg, Germany). Both institutions supported this project and kindly granted limited use of the necessary “House MD” episodes for our teaching purposes.

### Design of the seminars

Each seminar starts by showing a short intro scene of a specific “House MD” episode. Typically, the opening scene deals with the initial presentation of the patient (mostly rather acute, life-threatening situations) followed by a rather complex discussion on differential diagnosis within the team of Dr. House. At this point, we stop the video clip and switch to factual medical information and discuss the differential diagnosis at this point. Furthermore, most of the suggested diseases presented by the team of Dr. House were critically discussed with our students. We try to illuminate the value of each argument and clarify in the discussion with our students what is fact and what is fiction. Thereafter, we jump to the next interesting sequence of the TV episode. Here we usually see serious side effects of an initial treatment approach by Dr. House and his team. This again will be discussed in detail including patient safety aspects as well as undesired side effects. At the end of the episode, we see the final diagnosis by Dr. House and his team. This allows us to review and discuss the fact and fiction of the whole story critically. Finally, we summarize the correct approach in diagnosing the particular disease in the light of the current clinical practice. In total, a typical class includes 8 to 16 video clips of 1 to 2 minutes, taken from one particular “House MD” episode for a 90-minute seminar.

### Evaluation and statistical analysis of the seminar series

An external, independent evaluation was initiated from the beginning of this seminar in order to ensure quality and to detect the risk of a thinkable wrong role model early enough. As part of this evaluation, an anonymous 8-page questionnaire with 89 questions was completed by all participants once.

The questionnaire was comprised of three different types of questions. Most questions consist of Likert items assessing a statement on a 9-point scale from "I strongly agree" to "I do not agree at all". To ease graphical interpretation, the original 9-level Likert scale was condensed into 5 categories “Strong agreement” (5), “Agreement” (4), “Neutral” (3), “Disagreement” (2), “Strong disagreement” (1). In addition, the questionnaire included classical single choice and multiple-choice questions stating possible answers, of which one or several could be checked. The third type was open questions which could be answered in free text form by the students. The questionnaire asked the students for their motivation to visit this non-curricular seminar voluntarily, how they compared this seminar to traditional seminars, whether they received helpful knowledge and what they felt on Dr. House as a potential role model. Statistical analysis was performed using SAS Studio 3.5, University edition, SAS Institute, Heidelberg, Germany. Ordinal scaled Likert type data are given as median. Wilcoxon signed ranked test was used to assess statistical differences between the rating of the Dr. House Seminar and a traditional seminar. Kruskal-Wallis was used to determine if differences exist among students from different academic years on the seminar rating. A p-value below 0.05 was considered significant.

### Ethical approval

A formal waiver of ethical approval was granted by the Institutional Review Board (IRB) since this paper analyses anonymously received standard evaluation data from our medical students retrospectively and does not involve any studies in humans (EK-MR-17_08_17 Schäfer).

## Results

**Fig 1:**
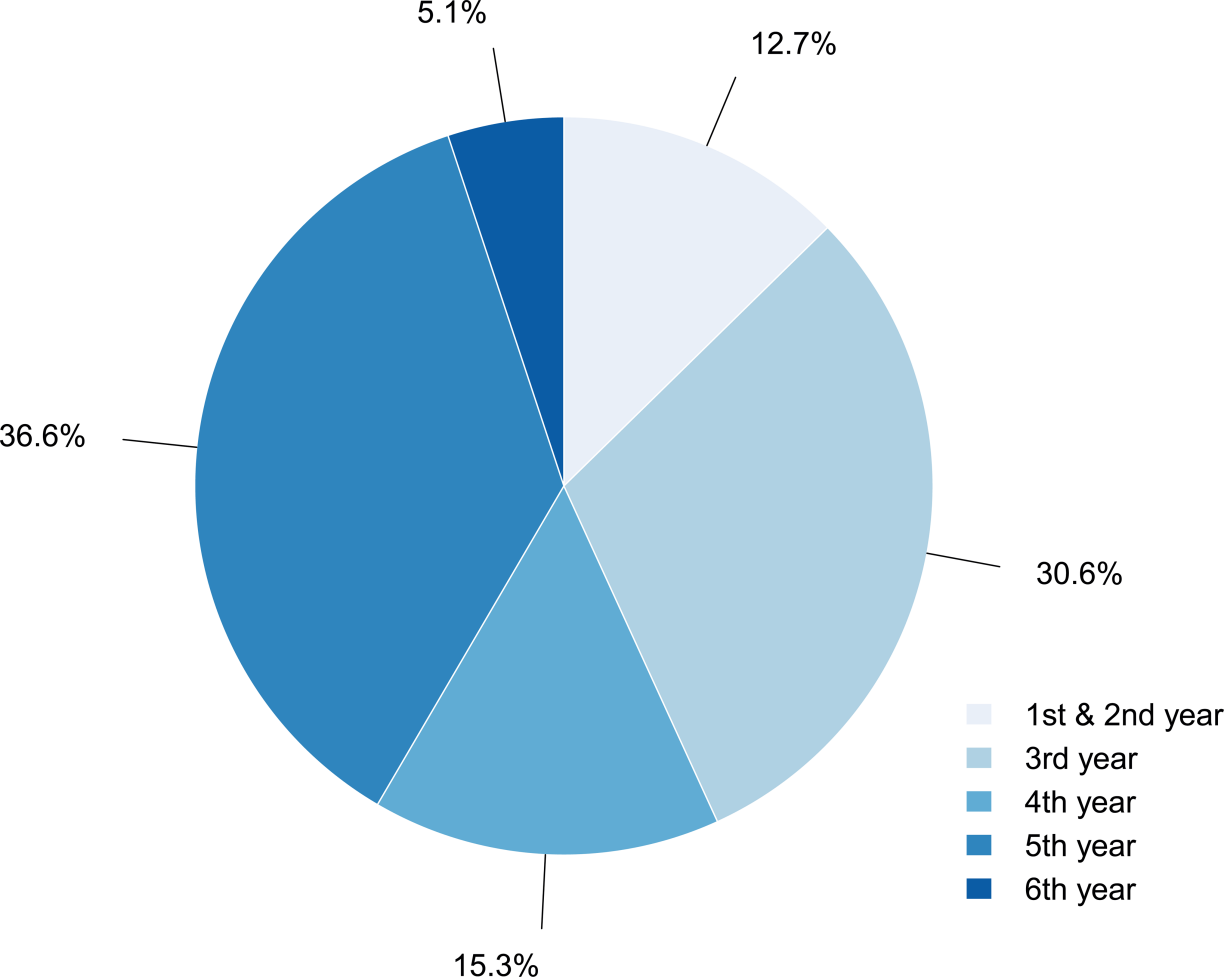
Percentage distribution of students by academic year.

Since autumn 2008 the seminars were held continuously with 6 to 8 sessions per academic semesters. Throughout this period, we collected 213 individual evaluations from students who participated regularly in the “House MD” seminar. Due to missing answers not for every question the full number of responses could be included in our assessment. Since this seminar was addressed to higher clinical medical students, most students were in their 5^th^ year (Fig 1).

### Why do students visit non-credited House MD seminars at all?

In the first year, the seminar took place on Saturday morning (09:00–10:30 o´clock). Later on, we switched to a Tuesday late afternoon (17:30–19:00 o´clock). The students do not receive any credits for their participation. Despite the rather student-unfriendly time schedule the seminar was well perceived and 20 to 30 students visited the seminar regularly.

**Fig 2:**
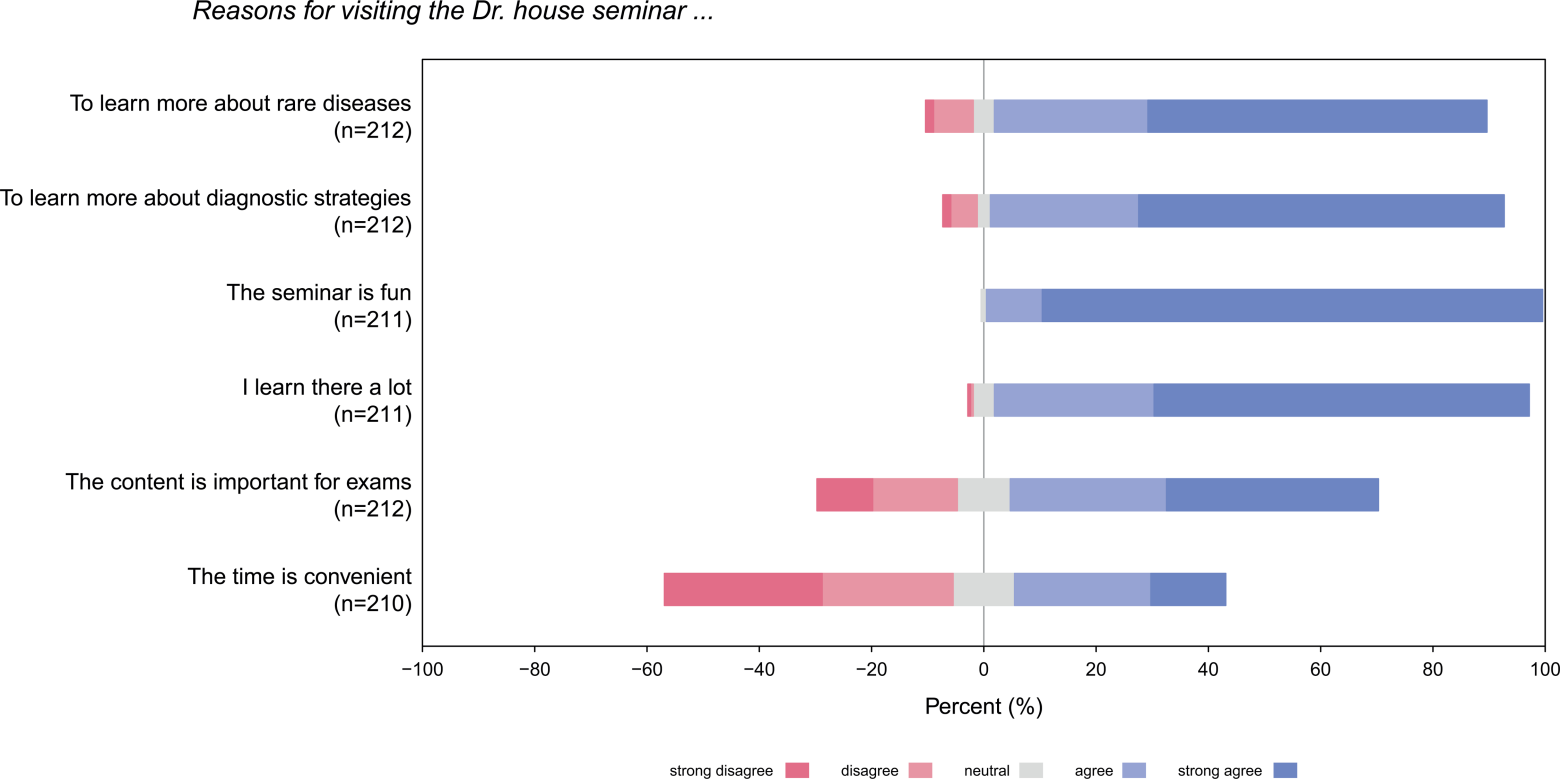
Reasons for visiting the Dr. House seminar.

When asked for different motivations for joining our seminar, the highest rated answers were “visiting this seminar makes fun”, “learning a lot”, and “learning more about diagnostic strategies”. In contrast, students did not participate to be better prepared for their final exams. Thus, students seem to come to this type of seminar for intrinsic reasons like fun and interest but less so for extrinsic reasons such as exams (Fig 2).

### How does “House MD” seminars compare to traditional seminars?

**Fig 3:**
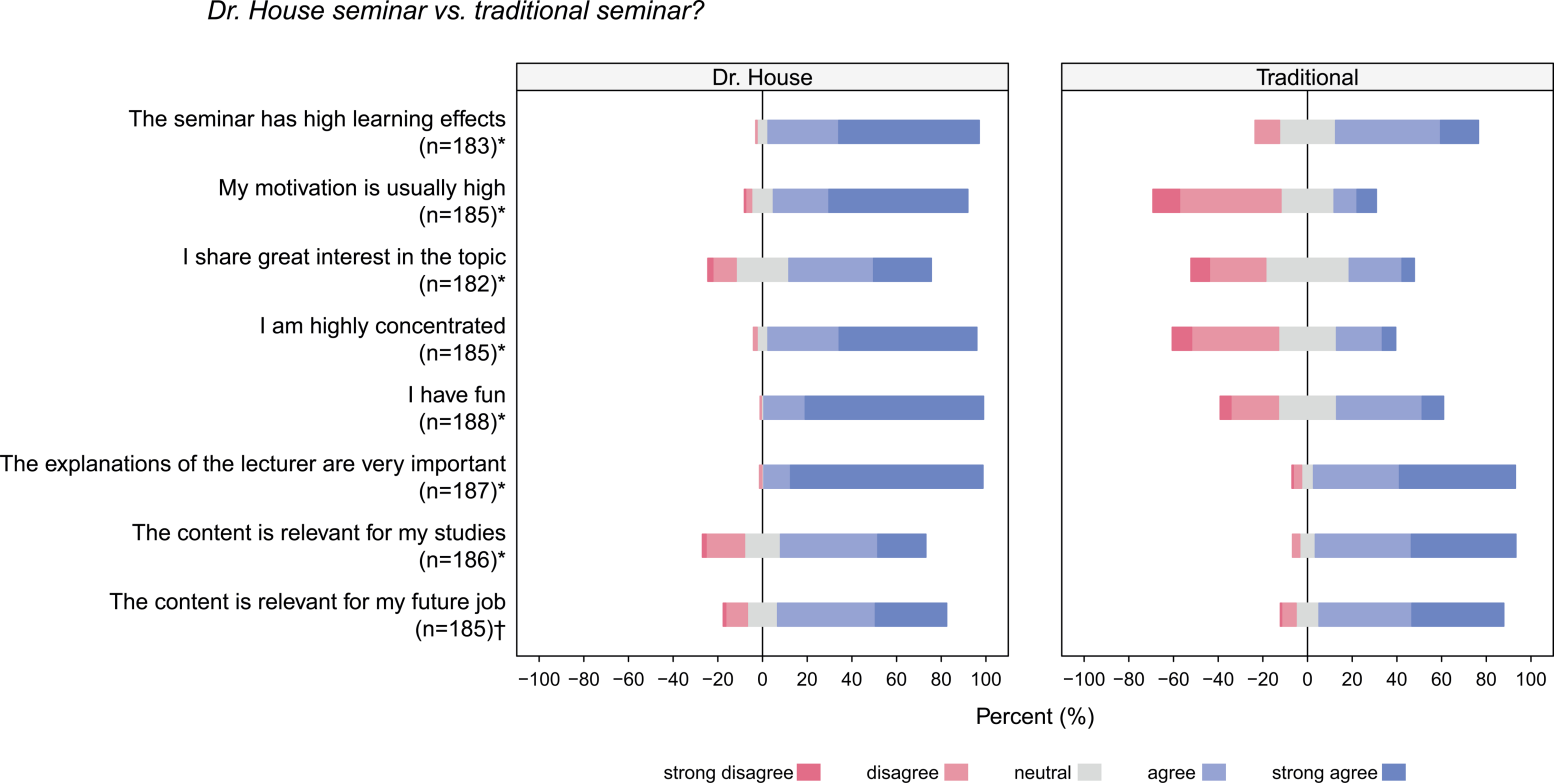
Comparison of Dr. House seminars to a traditional seminar. * Difference is significant p<0.001; † Difference is significant p<0.05.

One could argue that the students visiting “House MD” seminars were generally higher motivated and might enjoy all types of seminars. Therefore, we asked questions by which we compared the “House MD” seminar with traditional seminars. 69.9% of respondents reported higher learning effects (30.1% less). Motivation (89.7%), concentration (88.7%) and fun (86.7%) were higher rated by the majority, as well. The results are summarized in (Fig 3). Students consider the “House MD” seminar less essential for their studies or exams (p<0.001) and less essential for their later clinical work (p = 0.0298). However, students are more interested in the contents of the “House MD” seminars. Interestingly they are higher motivated and more concentrated compared to classical seminars. They feel to learn more since they memorize the contents better. In addition, the students find the “House MD” seminar to be more supportive in learning complex topics compared to classical seminars. Furthermore, they think it is more fun than regular seminars. At the same time, they feel that the lecturer is especially important in the “House MD” seminar to understand the content properly (all p<0.001). Despite these the students spend less time for reviewing a typical “House MD” seminar compared to standard seminars (14.8±24.3 minutes vs. 31.1±34.2 minutes; p<0.001). They also take more notes during regular seminars (0.4±0.7 pages vs. 1.2±1.3 pages; p<0.001). In this respect, the better performance of the “House MD” seminar is even more impressive. Worthwhile to note is the fact that the seminar ratings did not differ between students from different academic years (all p>0.05).

### Why is learning with “House MD” stimulating?

Our students are highly motivated, interested and think the seminar is fun. They describe this seminar as useful for learning complex medical contents. The students consider the role of the lecturer and his detailed explanations as very important,—more important than factual knowledge, diagnostic strategies, diseases explained, discussion with peers and recommended literature (see Table 1). Therefore, the strength of the “House MD” Seminar is in part the rather strong interactive involvement between the lecturers and the students.

**Table 1:** Contribution of key aspects to learning effect.

Contribution of key aspects to learning effect							
		(a)	(b)	(c)	(d)	(e)	(f)
**Knowledge of physicians in tv show**	**(a)**	-	0,128	-1.019*	-0.521*	-1.711*	0,169
**Diagnostic strategies of physicians in tv show**	**(b)**		-	-1.147*	-0.645*	-1.839*	0,034
**Diseases shown in the tv show**	**(c)**			-	0.490*	-0.695*	1.200*
**Discussions with classmates**	**(d)**				-	-1.190*	0.688*
**Explanations of lecturer**	**(e)**					-	1.874*
**Recommended literature**	**(f)**						-

Table illustrates the mean value of paired differences of key aspects to learning effects, as calculated by subtracting numeric Likert values. A positive value implicates a tendency towards the aspect stated in the row, a negative value towards the aspect mentioned in the column.

* values are significant p<0.05

### Does Dr. House influence our students as a role model?

**Fig 4:**
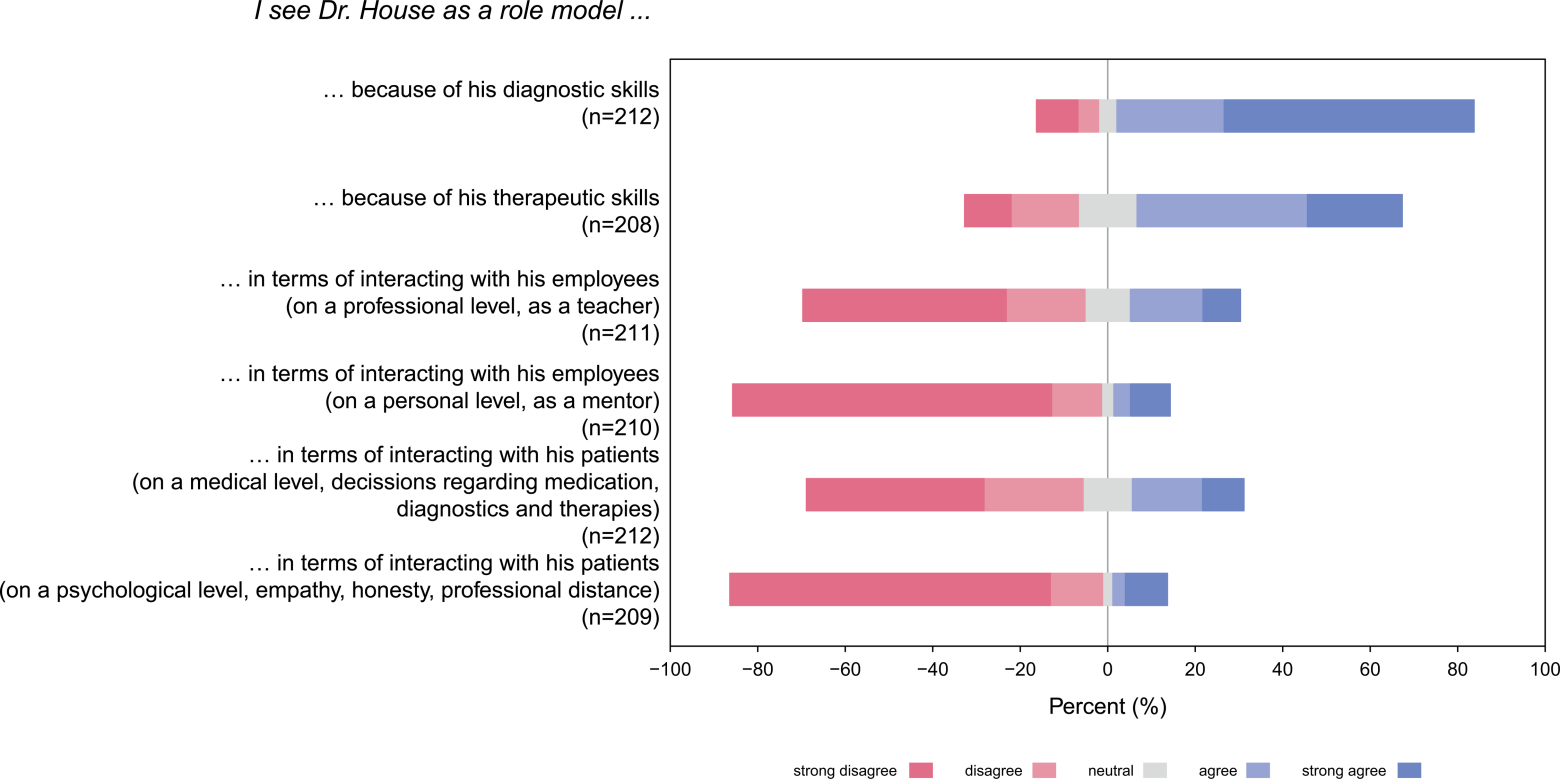
Evaluation of Dr. House as a potential role model.

Since Dr. House is a rather misanthropic character, one should be careful when using this TV series for teaching young students. Clearly, our students should not see this crude character as an acceptable role model. However, bringing Dr. House into the lecture hall, we are able to address and to control this critical issue. We can make clear statements as teachers—rather than leaving our students with this TV show alone. The students in our seminars differentiated extremely well between the diagnostic skills and the unacceptable interpersonal behavior of Dr. House. When asked if Dr. House could be seen as a role model in respect to his interaction with patients, agreement with those statements was generally low. A median of 2 was found for Dr. House´s interactions with his patients to find the appropriate medical decision (informed consent, diagnostic approach, therapy etc), and a median of 1 was found for his way of interacting with patients psychologically (honesty, empathy, professional distance). At the same time, the students appreciated his outstanding diagnostic and therapeutic capabilities (Fig 4).

### Viewing habits and influence of the House MD seminar?

The majority of our students watched medical dramas at home (n = 157; 76.6%). As only 64.2% (n = 129) watched medical dramas before entering medical school, these viewing habits changed significantly (p<0.001). Moreover, 55% (n = 110) stated that the “House MD” seminar increased their medical drama consumption and interestingly 71.5% (n = 143) said they would now “think more” when watching television shows. Only a minority (n = 27; 13.7%) stated that medical dramas influenced their decision to become a doctor.

## Discussion

In recent years, medical dramas such as “Emergency Room”, “Grey´s Anatomy” or “House MD” reached a broad viewership in the TV-watching western world. Besides pure entertainment, these series can and have been used for the purpose of medical education of the general public [4–6].

In the USA major efforts have been undertaken to ensure high-quality medical entertainment and to assist simultaneously public health activities. The National Institutes of Health (NIH, Bethesda, Maryland, USA) as well as the Center for Disease Control (CDC, Atlanta, Georgia USA) were supporting the "Norman Lear Center" in Los Angeles to assist filmmakers with scientific questions with the project "Hollywood, Health and Society" [7]. Martin Kaplan from the "Norman Lear Center" described the activities of his institute at the MINTiFF conference in 2010 [8] as a "use us as you choose" service for writers. Kaplan refers to „entertainment as an accidental curriculum”with an enormous „power of entertainment to affect people’s behavior“.

Besides the effects on the general population, medical dramas also influence the medical community in several ways. 3 out of 4 of our students stated to watch medical TV series. We need to be prepared to accompany and lead our young students to a better understanding and critical discussion of some of these series. Seeing how important our students rated the lecturer, we can assume that they know that TV series include both, fact and fiction and the lecturer is the one to tell them. Weaver and Wilson studied 386 undergraduate students' perceptions of professionalism, ethics, realism and role models by watching medical television programs such as **“**House MD”, “Scrubs”, and “Grey's Anatomy”. Students had high recall of ethical topics portrayed on the shows, and most believed that medical programs generally portrayed ideals of professionalism well. They found that medical programs offer considerable currency and relevance for students and may be useful in teaching strategies that engage students in ethical lessons on how to practice medicine [9,10]. In addition, Czarny et al systematically analyzed “Grey's Anatomy” and “House MD” for bioethical and professionalism contents. In both medical dramas, they found powerful portrayals of bioethical issues and deviations from the norms of professionalism. In contrast, exemplary depictions of professionalism were found to a much lesser degree in these two series [11]. Taken together there are good reasons to offer young students seminars where they can discuss ethical as well as medical issues of these TV series in more detail with experienced professionals.

For medical teaching, we can use only very few of the well-known TV-shows, for example “high quality” series, such as “House MD”, “Grey´s Anatomy” or “Emergency Room”. However, each of those formats has its own, distinct pedagogical value in certain areas. As Hirt et al. argue clips from "ER" and "Scrubs" offer numerous examples for teaching and learning. They consider "Grey's Anatomy" to be an ideal show to discuss ethics in medicine and to promote teamwork [5]. For us, most important is the medical content of these series. It is helpful that the “House MD” team feels almost morally obliged to a certain scientific and medical correctness. Katherine Lingenfelter, supervising producer of “House MD”, views it as a social responsibility in fictional series, such as “House MD”, to keep the scientific facts as correct as possible [12]. Of course these dramas main purpose has been one of entertainment and they are never completely representative of daily practice [13]. However, these characteristics could make the knowledge mediation more attractive.

For this the authors of “House MD” review medical journals and interesting case reports–including those reported in the NEJM, THE LANCET, BMJ and others. Since “House MD” is very well known by most of our students, they are attracted by the fact that we deliver medical information with a high proportion of entertainment. In general, it is recommended to establish a learning and teaching climate to make the learning experience fun [14,15]. Moreover, in a team context humor patterns are able to trigger positive socioemotional communication, procedural structure, and even new solutions [16]. Our teaching concept explicitly used the fun-factor of “House MD” to successfully “lure” students voluntarily into the lecture halls. Interestingly, this approach worked even for rather special and rare diseases in which they might otherwise not be interested. We were able to use the themes of this show to discuss rather rare diseases such as acute porphyria, Lyme disease, echinococcosis, Morbus Addison, Cushing´s disease, Erdheim-Chester Disease (see list of diagnosis in Wikipedia [17]).

It is fun to join these seminars, but furthermore the participants learn a lot about rare diseases they might have missed otherwise. Interestingly we diagnosed a patient with severe Cobalt intoxication (with cardiomyopathy, opticus-neuropathy, hearing loss, hypothyroidism, fever of unknown origin) due to a metal hip replacement which reminded us to a recent class where we used a comparable “House MD” episode (Season seven, episode eleven; Title: Family Practice). Although we must insist that we would have diagnosed this cobalt intoxication also without “Dr. House”. However, it is fair enough to say that for us the knowledge of this “House MD” episode was very helpful and speeded up our diagnostic approach dramatically. This unusual case report was published in “THE LANCET” entitled “Cobalt intoxication diagnosed with the help of Dr. House”. As expected this report received an enormous media attention and found itself among the top 5% of all research outputs ever scored by Altmetric [18–24]. Of course, the media attention was not at all for our clinical skills but purely for the door-opener “Dr. House” in our paper´s title. Due to this broad media attention, worldwide numerous patients with cobalt intoxications were diagnosed (in our center we identified a total of 3 with life-threatening intoxications and up to 10 with minor signs). Again, by this broad media coverage “Dr. House” was saving lives.

In respect to the most crucial question, if Dr. House might influence the behavior of our students, we are less concerned. We could show that our students see this character quite critically and not as a role model. In addition, the fact that we discuss some of his misbehavior extensively ensures a more critical approach from our students towards this crude character.

However, our study has some limitations. First, this seminar is voluntarily and might attract mostly students with high interest and motivation to learn more about rare diseases. By this, the rating for this seminar is positively biased. Second, all data shown are self-adjustments and objective final exams are missing. Nether the less the students like this type of teaching, are willing to learn on rare diseases and see the troublesome personality of Dr. House critically.

In summary, to the best of our knowledge, we are the first university hospital offering a systematic seminar for teaching rare diseases in internal medicine utilizing a very well-known medical TV show such as “House MD”. This is an important teaching approach as most studies confirm the potential of medical drama in teaching soft skills like teamwork, medical ethics and doctor-patient communication [25,6,26,9,27–29]. Interestingly, viewing episodes of “House MD” was almost a social event sometimes and many students discussed bioethical issues within the seminars, as reported by Czarny et al recently [11]. In addition, this seminar provided a perfect opening for frank, controversial discussions of medical and ethical issues. The seminar is extremely well perceived by our students and–most likely due to the cynical type of person of the protagonist–found an amazingly broad media coverage. If we want to compete in our lecture halls with more sophisticated teaching videos on a variety of different internet-based teaching platforms, we have to access new and interesting concepts by which we can lure our students into the lecture halls. For at least one thing we have in common with movie makers: none of us wants his audience to switch off.

## Supporting information

S1 File This is the questionnaire used in this study translated to English. (PDF)

S2 File This is the original questionnaire used in this study in German. (PDF)

S1 Table This is the dataset obtained from the participants with English headers. (XLSX)

S2 Table This is the original dataset obtained from the participants with German headers. (XLSX)
