# Supplementary material for: Teaching medicine with the help of “Dr. House”
Source: PLoS One. 2018 Mar 13;13(3):e0193972. doi: 10.1371/journal.pone.0193972 (PMC5849285; doi:10.1371/journal.pone.0193972)
Supplement: S1 File — (PDF) [file pone.0193972.s001.pdf]

Dear students of the seminar "Dr. House revisited - or: Did we also cured the patient in Marburg?"!

This seminar represents an innovation in the medical curriculum. Most innovations have advantages and disadvantages. With our survey we would like to know your opinion about this seminar. The survey is completely anonymous. It is important that you answer spontaneously and as honestly as possible.

Through your participation you will help to improve future medical teaching. For this we would like to thank you in advance for your support.!

Prof. Dr. Jürgen Schäfer, Endowed professorship for preventive cardiology, Universitätsklinikum Marburg, Philipps-Universität Marburg

Prof. Dr. Ulrich Glowalla, Research Group Instructions and Interactive Media (IIM), Fachbereich Psychologie, Justus-Liebig-Universität Gießen

**What are reasons for visiting Dr. House-seminar?**	Fully applicable								Does not apply at all
The contents are relevant to the examination.	☐	☐	☐	☐	☐	☐	☐	☐	☐
For me, the time (earlier evening) is convenient.	☐	☐	☐	☐	☐	☐	☐	☐	☐
To learn more about rare diseases.	☐	☐	☐	☐	☐	☐	☐	☐	☐
To learn more diagnostic strategies.	☐	☐	☐	☐	☐	☐	☐	☐	☐
I enjoy the event.	☐	☐	☐	☐	☐	☐	☐	☐	☐
I learn a lot there.	☐	☐	☐	☐	☐	☐	☐	☐	☐

**What do you think is the most important contribution to the learning effect?**	Fully applicable								Does not apply at all
The doctors' factual knowledge in the film	☐	☐	☐	☐	☐	☐	☐	☐	☐
Strategies of the doctors in the film	☐	☐	☐	☐	☐	☐	☐	☐	☐
Patients' clinical pictures in the film	☐	☐	☐	☐	☐	☐	☐	☐	☐
Discussions with fellow students during the film	☐	☐	☐	☐	☐	☐	☐	☐	☐
Explanation of the teacher during the film	☐	☐	☐	☐	☐	☐	☐	☐	☐
Additional technical literature	☐	☐	☐	☐	☐	☐	☐	☐	☐

The following questions refer to your learning behaviour in the Dr. House seminar. Also include the previous sessions in your response. Please try to answer as honestly as possible.

**How well do the following statements apply to you?**	Fully applicable								Does not apply at all
I think I can remember the contents of the Dr. House seminar very well.	☐	☐	☐	☐	☐	☐	☐	☐	☐
If the Dr. House seminar is cancelled, I'm happy to have a day off.	☐	☐	☐	☐	☐	☐	☐	☐	☐
After the Dr. House seminar I often discuss difficult areas with fellow students.	☐	☐	☐	☐	☐	☐	☐	☐	☐
My thoughts often drift off during the Dr. House seminar.	☐	☐	☐	☐	☐	☐	☐	☐	☐
I really enjoy attending the Dr. House seminar.	☐	☐	☐	☐	☐	☐	☐	☐	☐
The Dr. House Seminar is not suitable for the understanding of complex contents.	☐	☐	☐	☐	☐	☐	☐	☐	☐
I also like to interrupt a pleasant activity to go to the Dr. House seminar.	☐	☐	☐	☐	☐	☐	☐	☐	☐
If I am still with fellow students after the Dr. House seminar, we usually talk about other topics than the contents of the seminar.	☐	☐	☐	☐	☐	☐	☐	☐	☐
During the Dr. House seminar, I am fully focused all the time.	☐	☐	☐	☐	☐	☐	☐	☐	☐
The lecturer's explanations in the Dr. House seminar are very important to fully understand the material.	☐	☐	☐	☐	☐	☐	☐	☐	☐
The subject matter of the Dr. House seminar is very important for my further studies.	☐	☐	☐	☐	☐	☐	☐	☐	☐
The subject matter of the Dr. House seminar is very important for my future professional life.	☐	☐	☐	☐	☐	☐	☐	☐	☐

How long are your average transcripts of a Dr. House seminar session? ________ A4-Pages

How much time do you spend per week preparing for a Dr. House seminar? ________ Minutes

How much time do you spend per week with your follow-up to a Dr. House seminar? ________ Minutes

The Dr. House seminar represents an innovation in medical teaching. In order to compare it with traditional courses, we ask you on this page for your assessment of seminars as you know them from your previous studies. For the sake of simplicity, we would like to refer to the traditional events known to you as typical or normal seminars. Please try again to answer as honestly as possible.

**How well do the following statements apply to you?**	Fully applicable								Does not apply at all
I think I can remember the contents of the traditional seminar very well.	☐	☐	☐	☐	☐	☐	☐	☐	☐
If the traditional seminar is cancelled, I'm happy to have a day off.	☐	☐	☐	☐	☐	☐	☐	☐	☐
After the traditional seminar I often discuss difficult areas with fellow students.	☐	☐	☐	☐	☐	☐	☐	☐	☐
My thoughts often drift off during the traditional seminar.	☐	☐	☐	☐	☐	☐	☐	☐	☐
I really enjoy attending the traditional seminar.	☐	☐	☐	☐	☐	☐	☐	☐	☐
The traditional Seminar is not suitable for the understanding of complex contents.	☐	☐	☐	☐	☐	☐	☐	☐	☐
I also like to interrupt a pleasant activity to go to the traditional seminar.	☐	☐	☐	☐	☐	☐	☐	☐	☐
If I am still with fellow students after the traditional seminar, we usually talk about other topics than the contents of the seminar.	☐	☐	☐	☐	☐	☐	☐	☐	☐
During the traditional seminar, I am fully focused all the time.	☐	☐	☐	☐	☐	☐	☐	☐	☐
The lecturer's explanations in the traditional seminar are very important to fully understand the material.	☐	☐	☐	☐	☐	☐	☐	☐	☐
The subject matter of the traditional seminar is very important for my further studies.	☐	☐	☐	☐	☐	☐	☐	☐	☐
The subject matter of the traditional seminar is very important for my future professional life.	☐	☐	☐	☐	☐	☐	☐	☐	☐

How long are your average transcripts of a traditional seminar session? ________ A4-Pages

How much time do you spend per week preparing for a traditional seminar? ________ Minutes

How much time do you spend per week with your follow-up to a traditional seminar? ________ Minutes

**How well do the following statements correspond to your experience?**	Fully applicable								Does not apply at all
Discussions with fellow students sometimes show understanding for the fact that Dr. House behaves so abruptly towards patients in some situations.	☐	☐	☐	☐	☐	☐	☐	☐	☐
I'm worried that some of my fellow students might later become accustomed to behaving in a similar way to patients like Dr. House as doctors.	☐	☐	☐	☐	☐	☐	☐	☐	☐
Dr. House is a role model for many of my fellow students - this is how you set professional boundaries.	☐	☐	☐	☐	☐	☐	☐	☐	☐
I see the danger that prospective doctors will take Dr. House as a role model; however, the lecturer is very well prepared for this in his event.	☐	☐	☐	☐	☐	☐	☐	☐	☐
I don't think a doctor will behave as rudely as Dr. House will behave towards patients. But secretly, many fellow students think he's right.	☐	☐	☐	☐	☐	☐	☐	☐	☐
The way Dr. House treats patients is completely unacceptable to me and my fellow students.	☐	☐	☐	☐	☐	☐	☐	☐	☐
Dr. House's staff compensates very well for his interaction with the patients, so many of my fellow students don't find his behavior very bad.	☐	☐	☐	☐	☐	☐	☐	☐	☐

**For me Dr. House is a role model cause …**	Fully applicable								Does not apply at all
... in terms of his diagnostic skills	☐	☐	☐	☐	☐	☐	☐	☐	☐
... in terms of his therapeutic abilities	☐	☐	☐	☐	☐	☐	☐	☐	☐
... in terms of dealing with his employees (on a professional level, as their teacher)	☐	☐	☐	☐	☐	☐	☐	☐	☐
... in relation to dealing with his employees (on a personal level, as their boss)	☐	☐	☐	☐	☐	☐	☐	☐	☐
... in relation to the treatment of patients (medical: decisions on medication, diagnostics, operations...)	☐	☐	☐	☐	☐	☐	☐	☐	☐
... in relation to dealing with patients (psychological: honesty, rigour, professional distance, compassion...)	☐	☐	☐	☐	☐	☐	☐	☐	☐

**How much do you agree with the following statements?**	Fully applicable								Does not apply at all
I attend the seminar to learn more about rare diseases.	☐	☐	☐	☐	☐	☐	☐	☐	☐
I am much more interested in other aspects of the series than rare diseases.	☐	☐	☐	☐	☐	☐	☐	☐	☐
The illnesses described in the series are interesting, but due to their rarity they are not relevant for my later professional life.	☐	☐	☐	☐	☐	☐	☐	☐	☐
Since I started attending the seminar, I think that rare diseases are not treated enough in my studies.	☐	☐	☐	☐	☐	☐	☐	☐	☐
I have always found that rare diseases are not treated enough in my studies.	☐	☐	☐	☐	☐	☐	☐	☐	☐
My interest in rare diseases has increased considerably as a result of the seminar.	☐	☐	☐	☐	☐	☐	☐	☐	☐

For the following questions, you may find several answer alternatives to be correct. In this case, please check several answers.

Do you also watch medical series at home?	☐No	☐Yes, Dr. House	☐Ja, andere
Did you already watch medical series at home before the seminar?	☐No	☐Yes, Dr. House	☐Ja, andere
Have you already seen medical series at home before your studies?	☐No	☐Yes, Dr. House	☐Ja, andere
Have doctor series influenced your choice of profession?	☐No	☐Yes, in deciding to become a doctor	☐ Yes, when deciding on a speciality
If so, which series / doctors did you influence?			
Has your participation in the Dr. House seminar changed the way you think during the series?	☐ No	☐ Yes, quantitati ve (more)	☐ Yes, qualitatively (higher value)

Do you know of any other TV shows or films that could be used to create similar seminars? If yes, which ones?	
Can you think of other sources of information or media that could be used to design similar seminars? If yes, which ones?	

In your opinion, does the lecturer have a different role or function in this seminar than in other seminars?

□Yes □Other role □Other function

If yes, please describe the changes briefly:	

How important is the lecturer in the Dr. House seminar for the quality of teaching?

□ *Less important than in other seminars* □ *Just as important as in other seminars* □ *More important than in other seminars*

Which aspects of the Dr. House seminar did you particularly like?	
Which aspects of the Dr. House seminar did you most disturb?	
Would you like to tell us something else that has not been mentioned in any of the questions?	

How did you hear about the Dr. House seminar?

☐ Media ☐ Fellow students ☐ Course-cataloge ☐ Other: ________

Have you attended the Dr. House seminar in the last semester?

☐ yes ☐ no

Which semester are you currently attending? ________Semester

How often did you attend the Dr. House seminar?

☐ 1x ☐ 2-5 ☐ 5-10 ☐ 10-15

Please assess how well the following statements apply to the Dr. House seminar from your point of view!

	Fully applicable								Does not apply at all
Learning in the Dr. House seminar is fun.	☐	☐	☐	☐	☐	☐	☐	☐	☐
The medial representation makes it easier to remember the material.	☐	☐	☐	☐	☐	☐	☐	☐	☐
The lecturer is didactically and professionally very good.	☐	☐	☐	☐	☐	☐	☐	☐	☐
Differential diagnostics and the diagnostic process are discussed in detail.	☐	☐	☐	☐	☐	☐	☐	☐	☐
The atmosphere is more relaxed and learning friendly than in a regular seminar.	☐	☐	☐	☐	☐	☐	☐	☐	☐
The interesting and instructive material has increased my motivation.	☐	☐	☐	☐	☐	☐	☐	☐	☐
The cases are explained on the basis of "real" patients and are therefore more complex than "pure" clinical pictures.	☐	☐	☐	☐	☐	☐	☐	☐	☐
Socially critical topics with regard to the treatment of patients is discussed.	☐	☐	☐	☐	☐	☐	☐	☐	☐
We students are constantly encouraged to actively engage ourselves.	☐	☐	☐	☐	☐	☐	☐	☐	☐

Thank you for your participation!
