# Supplementary material for: Teaching medicine with the help of “Dr. House”
Source: PLoS One. 2018 Mar 13;13(3):e0193972. doi: 10.1371/journal.pone.0193972 (PMC5849285; doi:10.1371/journal.pone.0193972)
Supplement: S2 File — (PDF) [file pone.0193972.s002.pdf]

Liebe Studierenden des Seminars "Dr. House revisited - oder: Hätten wir den Patienten in Marburg auch geheilt?"!

Dieses Seminar stellt eine Innovation im medizinischen Lehrplan dar. Die meisten Innovationen haben Vor- und Nachteile. Mit unserer Befragung möchten wir Ihre Meinung zu diesem Seminar erfahren. Die Befragung ist vollständig anonym. Es ist wichtig, dass Sie spontan und so ehrlich wie möglich antworten.

Durch Ihre Teilnahme helfen Sie mit, zukünftige medizinische Lehre zu verbessern. Hierfür bereits im Voraus unseren herzlichen Dank!

Prof. Dr. Jürgen Schäfer, Stiftungsprofessur für Präventive Kardiologie, Universitätsklinikum Marburg, Philipps-Universität Marburg

Prof. Dr. Ulrich Glowalla, Forschungsgruppe Instruktion und Interaktive Medien (IIM), Fachbereich Psychologie, Justus-Liebig-Universität Gießen

**Was sind Gründe dafür, dass Sie das Dr. House-Seminar besuchen?**	Trifft voll zu								Trifft gar nicht zu
Die Inhalte sind prüfungsrelevant.	❑	❑	❑	❑	❑	❑	❑	❑	❑
Der Zeitpunkt (früher Abend) ist für mich günstig.	❑	❑	❑	❑	❑	❑	❑	❑	❑
Um mehr über seltene Erkrankungen zu lernen.	❑	❑	❑	❑	❑	❑	❑	❑	❑
Um mehr diagnostische Strategien kennen zu lernen.	❑	❑	❑	❑	❑	❑	❑	❑	❑
Die Veranstaltung macht mir Spaß.	❑	❑	❑	❑	❑	❑	❑	❑	❑
Ich lerne dort sehr viel.	❑	❑	❑	❑	❑	❑	❑	❑	❑

**Was trägt Ihrer Meinung nach am meisten zum Lerneffekt bei?**	Trifft voll zu								Trifft gar nicht zu
Faktenwissen der Ärzte im Film	❑	❑	❑	❑	❑	❑	❑	❑	❑
Strategien der Ärzte im Film	❑	❑	❑	❑	❑	❑	❑	❑	❑
Krankheitsbilder der Patienten im Film	❑	❑	❑	❑	❑	❑	❑	❑	❑
Diskussionen mit Kommilitonen während des Films	❑	❑	❑	❑	❑	❑	❑	❑	❑
Erläuterungen des Dozenten während des Films	❑	❑	❑	❑	❑	❑	❑	❑	❑
Zusätzliche Fachliteratur	❑	❑	❑	❑	❑	❑	❑	❑	❑

Die folgenden Fragen beziehen sich auf Ihr Lernverhalten im Dr. House-Seminar. Beziehen Sie auch die bereits vergangenen Sitzungen in Ihre Antwort mit ein. Bitte versuchen Sie, so ehrlich wie möglich zu antworten.

**Wie gut treffen die folgenden Aussagen auf Sie zu?**	Trifft voll zu								Trifft gar nicht zu
Ich glaube, dass ich mir die im Dr. House-Seminar behandelten Inhalte sehr gut merken kann.	❑	❑	❑	❑	❑	❑	❑	❑	❑
Wenn das Dr. House-Seminar einmal ausfällt, freue ich mich, dass ich frei habe.	❑	❑	❑	❑	❑	❑	❑	❑	❑
Häufig bespreche ich nach dem Dr. House-Seminar schwierige Stellen mit Kommilitonen.	❑	❑	❑	❑	❑	❑	❑	❑	❑
Meine Gedanken schweifen während des Dr. House-Seminars häufig ab.	❑	❑	❑	❑	❑	❑	❑	❑	❑
Der Besuch des Dr. House-Seminars macht mir richtig Spaß.	❑	❑	❑	❑	❑	❑	❑	❑	❑
Für das Verständnis komplexer Inhalte ist das Dr. House-Seminar nur schlecht geeignet.	❑	❑	❑	❑	❑	❑	❑	❑	❑
Ich unterbreche auch gern eine angenehme Tätigkeit, um zum Dr. House-Seminar zu gehen.	❑	❑	❑	❑	❑	❑	❑	❑	❑
Wenn ich nach dem Dr. House-Seminar noch mit Kommilitonen zusammenstehe, dann sprechen wir meist über andere Themen als die Inhalte des Seminars.	❑	❑	❑	❑	❑	❑	❑	❑	❑
Während des Dr. House-Seminars bin ich die ganze Zeit voll konzentriert.	❑	❑	❑	❑	❑	❑	❑	❑	❑
Die Erläuterungen des Dozenten sind im Dr. House-Seminar sehr wichtig, um den Stoff vollständig zu verstehen.	❑	❑	❑	❑	❑	❑	❑	❑	❑
Der Stoff des Dr. House-Seminars ist sehr wichtig für mein weiteres Studium.	❑	❑	❑	❑	❑	❑	❑	❑	❑
Der Stoff des Dr. House-Seminars ist sehr wichtig für mein späteres Berufsleben.	❑	❑	❑	❑	❑	❑	❑	❑	❑

Wie lang sind durchschnittlich Ihre Mitschriften einer Sitzung des Dr. House-Seminars? ________ A4-Seiten

Wie viel Zeit verbringen Sie pro Woche mit Ihrer *Vorbereitung* auf ein Dr. House-Seminar? ________ Minuten

Wie viel Zeit verbringen Sie pro Woche mit Ihrer *Nachbereitung* eines Dr. House-Seminars? ________ Minuten

Das Dr. House-Seminar stellt eine Innovation in der medizinischen Lehre dar. Um es mit traditionellen Lehrveranstaltungen vergleichen zu können, bitten wir Sie auf dieser Seite um Ihre Einschätzung zu Seminaren, wie Sie sie aus Ihrem bisherigen Studium kennen. Der Einfachheit halber bezeichnen wir die herkömmlichen, Ihnen bekannten Veranstaltungen im Folgenden als typisches oder normales Seminar. Bitte versuchen Sie wieder, so ehrlich wie möglich zu antworten.

**Wie gut treffen die folgenden Aussagen auf Sie zu?**	Trifft voll zu								Trifft gar nicht zu
Ich glaube, dass ich mir die in einem typischen Seminar behandelten Inhalte sehr gut merken kann.	❑	❑	❑	❑	❑	❑	❑	❑	❑
Wenn ein normales Seminar einmal ausfällt, freue ich mich, dass ich frei habe.	❑	❑	❑	❑	❑	❑	❑	❑	❑
Häufig bespreche ich nach einem typischen Seminar schwierige Stellen mit Kommilitonen.	❑	❑	❑	❑	❑	❑	❑	❑	❑
Meine Gedanken schweifen während eines typischen Seminars häufig ab.	❑	❑	❑	❑	❑	❑	❑	❑	❑
Der Besuch eines normalen Seminars macht mir richtig Spaß.	❑	❑	❑	❑	❑	❑	❑	❑	❑
Für das Verständnis komplexer Inhalte ist ein normales Seminar nur schlecht geeignet.	❑	❑	❑	❑	❑	❑	❑	❑	❑
Ich unterbreche auch gern eine angenehme Tätigkeit, um zu einem typischen Seminar zu gehen.	❑	❑	❑	❑	❑	❑	❑	❑	❑
Wenn ich nach einem normalen Seminar noch mit Kommilitonen zusammenstehe, dann sprechen wir meist über andere Themen als die Inhalte des Seminars.	❑	❑	❑	❑	❑	❑	❑	❑	❑
Während eines typischen Seminars bin ich die ganze Zeit voll konzentriert.	❑	❑	❑	❑	❑	❑	❑	❑	❑
Die Erläuterungen des Dozenten sind in einem typischen Seminar sehr wichtig, um den Stoff vollständig zu verstehen.	❑	❑	❑	❑	❑	❑	❑	❑	❑
Der Stoff eines typischen Seminars ist sehr wichtig für mein weiteres Studium.	❑	❑	❑	❑	❑	❑	❑	❑	❑
Der Stoff eines typischen Seminars ist sehr wichtig für mein späteres Berufsleben.	❑	❑	❑	❑	❑	❑	❑	❑	❑

Wie lang sind durchschnittlich Ihre Mitschriften einer Sitzung eines typischen Seminars? ________ A4-Seiten

Wie viel Zeit verbringen Sie pro Woche mit Ihrer *Vorbereitung* auf ein typisches Seminar? ________ Minuten

Wie viel Zeit verbringen Sie pro Woche mit Ihrer *Nachbereitung* eines typischen Seminars? ________ Minuten

**Wie sehr entsprechen die folgenden Aussagen Ihrer Erfahrung?**	Trifft voll zu								Trifft gar nicht zu
In Gesprächen mit Kommilitonen zeigt sich mitunter Verständnis dafür, dass sich Dr. House in manchen Situationen Patienten gegenüber so schroff verhält.	❑	❑	❑	❑	❑	❑	❑	❑	❑
Ich mache mir Sorgen, dass einige meiner Kommilitonen sich vielleicht später als Ärzte ein ähnliches Verhalten gegenüber Patienten wie Dr. House angewöhnen könnten.	❑	❑	❑	❑	❑	❑	❑	❑	❑
Dr. House ist für viele meiner Kommilitonen ein Vorbild – so setzt man professionelle Grenzen.	❑	❑	❑	❑	❑	❑	❑	❑	❑
Ich sehe die Gefahr, dass angehende Ärzte sich Dr. House als Rollenvorbild nehmen; allerdings fängt der Dozent das in seiner Veranstaltung sehr gut auf.	❑	❑	❑	❑	❑	❑	❑	❑	❑
Ganz so grob wie Dr. House wird sich wohl kein Arzt gegenüber Patienten verhalten. Aber insgeheim finden viele Kommilitonen, dass er damit recht hat.	❑	❑	❑	❑	❑	❑	❑	❑	❑
Die Art, in der Dr. House mit Patienten umgeht, ist für mich und meine Kommilitonen vollständig inakzeptabel.	❑	❑	❑	❑	❑	❑	❑	❑	❑
Die Mitarbeiter von Dr. House kompensieren seinen Umgang mit den Patienten sehr gut; daher finden viele meiner Kommilitonen sein Verhalten nicht weiter schlimm.	❑	❑	❑	❑	❑	❑	❑	❑	❑

**Für mich ist Dr. House ein Vorbild…**	Trifft voll zu								Trifft gar nicht zu
… in Bezug auf seine diagnostischen Fähigkeiten	❑	❑	❑	❑	❑	❑	❑	❑	❑
… in Bezug auf seine therapeutischen Fähigkeiten	❑	❑	❑	❑	❑	❑	❑	❑	❑
… in Bezug auf den Umgang mit seinen Mitarbeitern (auf fachlicher Ebene, als ihr Lehrer)	❑	❑	❑	❑	❑	❑	❑	❑	❑
… in Bezug auf den Umgang mit seinen Mitarbeitern (auf persönlicher Ebene, als ihr Chef)	❑	❑	❑	❑	❑	❑	❑	❑	❑
… in Bezug auf den Umgang mit Patienten (medizinisch: Entscheidungen über Medikation, Diagnostik, Operationen…)	❑	❑	❑	❑	❑	❑	❑	❑	❑
… in Bezug auf den Umgang mit Patienten (psychologisch: Ehrlichkeit, Strenge, professionelle Distanz, Mitgefühl…)	❑	❑	❑	❑	❑	❑	❑	❑	❑

**Wie sehr stimmen Sie folgenden Aussagen zu?**	Trifft voll zu								Trifft gar nicht zu
Ich besuche das Seminar, um mehr über seltene Krankheiten zu lernen.	❑	❑	❑	❑	❑	❑	❑	❑	❑
Mich interessieren andere Aspekte der Serie wesentlich mehr als die seltenen Erkrankungen.	❑	❑	❑	❑	❑	❑	❑	❑	❑
Die Erkrankungen, die in der Serie dargestellt werden, sind zwar interessant, aber wegen ihrer Seltenheit wenig relevant für mein späteres Berufsleben.	❑	❑	❑	❑	❑	❑	❑	❑	❑
Seit ich das Seminar besuche, denke ich, dass seltene Krankheiten im Studium zu wenig behandelt werden.	❑	❑	❑	❑	❑	❑	❑	❑	❑
Ich fand schon immer, dass seltene Krankheiten im Studium zu wenig behandelt werden.	❑	❑	❑	❑	❑	❑	❑	❑	❑
Mein Interesse an seltenen Erkrankungen ist durch das Seminar erheblich gestiegen.	❑	❑	❑	❑	❑	❑	❑	❑	❑

Bei den folgenden Fragen kommen Ihnen vielleicht mehrere Antwortalternativen richtig vor. Bitte kreuzen Sie in diesem Fall ruhig mehrere Antworten an.

Sehen Sie sich auch zuhause Arztserien an?	❑Nein	❑Ja, Dr. House	❑Ja, andere
Haben Sie bereits vor dem Seminar zuhause Arztserien angesehen?	❑Nein	❑Ja, Dr. House	❑Ja, andere
Haben Sie bereits vor dem Studium zuhause Arztserien gesehen?	❑Nein	❑Ja, Dr. House	❑Ja, andere
Haben Arztserien Sie bei der Berufswahl beeinflusst?	❑Nein	❑Ja, bei der Entscheidung, Arzt zu werden	❑Ja, bei der Entscheidung für einen bestimmten Schwerpunkt
Wenn ja, welche Serie / welche Ärzte haben Sie beeinflusst?			
Hat sich durch Ihre Teilnahme am Dr. House-Seminar die Art verändert, wie Sie während der Serie „mitdenken"?	❑ Nein	❑ Ja, quantitativ (mehr)	❑Ja, qualitativ (höherwertig)

Kennen Sie weitere Fernsehsendungen oder Filme, mit denen sich ähnliche Seminare gestalten ließen? Wenn ja, welche?			
Fallen Ihnen weitere Informationsquellen oder Medien ein, mit denen sich ähnliche Seminare gestalten ließen? Wenn ja, welche?			
Hat der Dozent Ihrer Meinung nach in diesem Seminar eine andere Rolle oder eine andere Funktion als in anderen Seminaren?	❑Nein	❑Eine andere Rolle	❑Eine andere Funktion
Wenn ja, dann beschreiben Sie die Veränderungen bitte kurz:			
Wie wichtig ist der Dozent im Dr. House-Seminar für die Qualität der Lehre?	❑*Weniger wichtig* als in anderen Seminaren	❑*Genauso wichtig* wie in anderen Seminaren	❑*Wichtiger* als in anderen Seminaren
Welche Aspekte des Dr. House-Seminars haben Ihnen besonders gut gefallen?			
Welche Aspekte des Dr. House-Seminars haben Sie am meisten gestört?			
Möchten Sie uns noch etwas mitteilen, was in keiner der Fragen angesprochen wurde?			

Wie sind sie auf das Dr. House-Seminar aufmerksam geworden?

❑ Medien ❑ Kommilitonen ❑ Vorlesungsverzeichnis ❑ Sonstiges: ________

Haben Sie das Dr. House-Seminar schon im letzten Semester besucht?

❑ ja ❑ nein

Welches Semester besuchen Sie derzeit? ________ Semester

Wie oft haben Sie in etwa das Dr. House-Seminar besucht?

❑ 1x ❑ 2-5 ❑ 5-10 ❑ 10-15

Bitte beurteilen Sie, wie gut die folgenden Aussagen aus Ihrer Sicht auf das Dr. House-Seminar zutreffen!

	Trifft voll zu								Trifft gar nicht zu
Das Lernen im Dr. House-Seminar macht Spaß.	❑	❑	❑	❑	❑	❑	❑	❑	❑
Durch die mediale Darstellung fällt es leichter, sich den Stoff zu merken.	❑	❑	❑	❑	❑	❑	❑	❑	❑
Der Dozent ist didaktisch und fachlich sehr gut.	❑	❑	❑	❑	❑	❑	❑	❑	❑
Die Differentialdiagnostik und der Diagnoseprozess werden ausführlich besprochen.	❑	❑	❑	❑	❑	❑	❑	❑	❑
Die Atmosphäre ist lockerer und lernfreundlicher als in einem regulären Seminar.	❑	❑	❑	❑	❑	❑	❑	❑	❑
Der interessante und lehrreiche Stoff hat meine Motivation erhöht.	❑	❑	❑	❑	❑	❑	❑	❑	❑
Die Fälle werden anhand von „wirklichen“ Patienten erklärt und sind daher komplexer als „reine“ Krankheitsbilder.	❑	❑	❑	❑	❑	❑	❑	❑	❑
Auch sozialkritische Themen im Hinblick auf die Behandlung von Patienten werden besprochen.	❑	❑	❑	❑	❑	❑	❑	❑	❑
Wir Studierende werden immer wieder ermuntert, uns selbst aktiv einzubringen.	❑	❑	❑	❑	❑	❑	❑	❑	❑

Vielen Dank für Ihre Mitarbeit!
